# Superanionic Solvent‐Free Liquid Enzymes Exhibit Enhanced Structures and Activities

**DOI:** 10.1002/advs.202202359

**Published:** 2022-08-21

**Authors:** Ye Zhou, Jannik Nedergaard Pedersen, Jacob Nedergaard Pedersen, Nykola C. Jones, Søren Vrønning Hoffmann, Steen Vang Petersen, Jan Skov Pedersen, Adam Perriman, Renjun Gao, Zheng Guo

**Affiliations:** ^1^ Key Laboratory for Molecular Enzymology and Engineering The Ministry of Education School of Life Sciences Jilin University No. 2699, Qianjin Street Changchun 130012 P. R. China; ^2^ Department of Biological and Chemical Engineering Aarhus University Gustav Wieds Vej 10 Aarhus 8000 Denmark; ^3^ Department of Chemistry and Interdisciplinary Nanoscience Center (iNANO) Aarhus University Gustav Wieds Vej 14 Aarhus 8000 Denmark; ^4^ ISA Department of Physics and Astronomy Aarhus University Ny Munkegade 120 Aarhus 8000 Denmark; ^5^ Department of Biomedicine Aarhus University Wilhelm Meyers Allé 4 Aarhus 8000 Denmark; ^6^ School of Cellular and Molecular Medicine University of Bristol BS8 1TS Bristol UK

**Keywords:** anionization, cationic polymer surfactant, lipase, myoglobin, solvent‐free liquid enzyme

## Abstract

The surface of a carboxylate‐enriched octuple mutant of *Bacillus subtilis* lipase A (8M) is chemically anionized to produce core (8M)‐shell (cationic polymer surfactants) bionanoconjugates in protein liquid form, which are termed anion‐type biofluids. The resultant lipase biofluids exhibit a 2.5‐fold increase in hydrolytic activity when compared with analogous lipase biofluids based on anionic polymer surfactants. In addition, the applicability of the anion‐type biofluid using Myoglobin (Mb) that is well studied in anion‐type solvent‐free liquid proteins is evaluated. Although anionization resulted in the complete unfolding of Mb, the active *α*‐helix level is partially recovered in the anion‐type biofluids, and the effect is accentuated in the cation‐type Mb biofluids. These highly active anion‐type solvent‐free liquid enzymes exhibit increased thermal stability and provide a new direction in solvent‐free liquid protein research.

## Graphical abstract

Novel cationic polymer surfactant is synthesized and applied for the preparation of anionic solvent‐free liquid lipases and myoglobin. The anionic lipase biofluids exhibit a 2.5‐fold increase in hydrolytic activity compared with analogous anionic lipase biofluids. For myoglobin enhanced structure is observed upon being electrostatically conjugated with cationic polymer surfactants following anionization. This work provides a new direction in solvent‐free liquid protein research to obtain highly active thermostable enzyme biofluids.

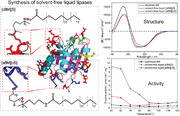

## 1 Introduction

Solvent‐free liquid proteins (also referred to as biofluids or protein liquids) are a new class of hybrid nano‐biomaterials,^[^ ^1^ ^]^ and have potential in biocatalysis for some of their striking properties, including near‐native structure, hyper‐thermal stability, and reversible folding.^[^ ^2^, ^3^ ^]^ To date, the synthetic pathway to produce the solvent‐free liquids has involved cationic supercharging of the protein surface, followed by electrostatic conjugation using anionic polymer surfactants, which if followed by lyophilization and thermal annealing to anhydrous protein liquids. This has been successfully applied to both commercially available enzymes (e.g., ferritin,^[^ ^4^ ^]^ myoglobin,^[^ ^2^, ^5^ ^]^ lipase,^[^ ^3^ ^]^ etc.) and genetically engineered enzymes (i.e., *Bacillus subtilis* lipase A (BsLA)^[^ ^6^ ^]^). However, proteins with naturally high levels of surface carboxylates are likely to lose activity after cationization and polymer conjugation, especially if some of the modifiable carboxylates are in the active site region. For example, the lipase from *Thermomyces lanuginosus* (TLL) is rich in carboxylates, both on the surface and near the active site region, and loses more than 90% of its initial hydrolytic activity after cationization, and polymer surfactant conjugation results in further loss in activity.^[^ ^3^ ^]^ Moreover, in our previous study, the mutants of BsLA that contained an increase in the number of surface carboxylates showed lower activity in protein liquids than the wild‐type BsLA, which exhibits no charged residues around the active sites.^[^ ^6^ ^]^

It was previously reported that a fluid material with high protein content (120 – 310 mg mL^–1^) could be formed through the ordered self‐assembly of native proteins segregated from water.^[^ ^7^ ^]^ Here, the material was instantly prepared by the simple mixing of a native protein solution with anionic and cationic surfactants, the molar ratio of which was optimized according to protein isoelectric point.^[^ ^7^ ^]^ Accordingly, proteins were assumed to alternatively form anion‐type protein liquids via anionization and subsequent conjugation of cationic polymer surfactants, especially, with much less loss in activity.

## 2 Result

**Figure 1:**
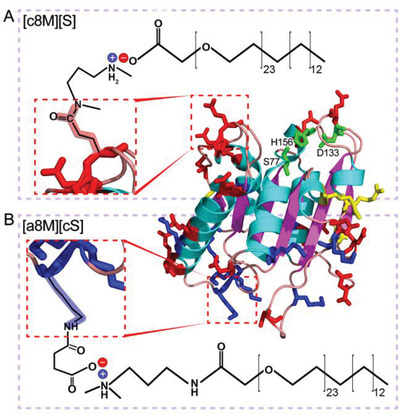
Graphic showing the carbodiimide‐mediated cationization of a glutamic acid side chain with N,N’‐dimethyl‐1,3‐propanediamine (DMPA) to yield c8M (or cMb), followed by electrostatic coupling with anionic polymer surfactants (S) to give [c8M][S] (or [cMb][S]) (A); and the succinylation of a lysine side chain with succinic anhydride to yield a8M (or aMb), followed by electrostatic conjugation of cationic polymer surfactants (cS) to give [a8M][cS] (or [aMb][cS]) (B). Surface carboxyl amino acids, lysine, and arginine are marked with red, blue, and yellow sticks, respectively. The active sites of 8M are marked in green.

**Synthesis of anion‐type and cation‐type biofluids**. Protein anionization commonly involves the modification of amine residues (lysine) by acylation with anhydrides (i.e., succinic anhydride), which not only eliminates the positive charge contribution of the protonated amine, but also adds the negative charge contribution of the acid, resulting in a change of minus two in net charge per group modified. Modification of surface charge by succinylation has been reported to render enzymes tolerance to ionic liquids (i.e., [BMIM][Cl]), which are excellent solvents for anhydrous biocatalysis.^[^ ^8^, ^9^ ^]^ Contrary to lysine, arginine cannot be reacted by succinic anhydride and will remain repulsive force against the conjugated polymers. In order to minimize this negative factor, a previously studied octuple mutant of BsLA (8M: F17E, A20E, K44E, Y49E, R57E, G111D, M134E, and G158E) that contains only four arginine residues was selected as a model enzyme in the present study (**Figure** **1**).^[^ ^10^ ^]^ Structurally, 8M shows a compact *α*/*β*‐hydrolase fold with no “lid” domain covering the active site (Ser77‐His156‐Asp133),^[^ ^11^, ^12^ ^]^ leaving a less hydrophobic patch with the genetically introduced carboxylates (Figure 1), which may contribute to largely improved solubility of the enzyme.^[^ ^10^ ^]^ Carboxylated Brij L23 was used as the anionic polymer surfactant (S),^[^ ^3^, ^13^ ^]^ and the cationic polymer surfactant (cS) was synthesized via condensation between 3‐dimethylaminopropylamine and carboxylated Brij L23 as described before (Figures S3 and S4, Supporting Information).^[^ ^7^ ^]^ Furthermore, we evaluated the applicability of the anion‐type biofluids using myoglobin (Mb), which has been well studied in terms of the cation‐type protein liquid (Figure 1).^[^ ^2^, ^5^, ^14^ ^]^

**Figure 2:**
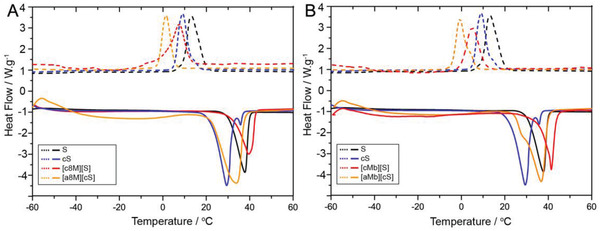
Cyclic DSC thermograms showing endothermic melting transitions (solid lines) and exothermic crystallization transitions (dashed lines) for 8M‐based A) and Mb‐based B) biofluids. The DSC curves of anionic (S) and cationic (cS) surfactants are marked in black and blue, respectively.

For a parallel comparison, the purified 8 M and Mb were then used as the precursors for both cation‐type and anion‐type biofluid synthesis (see the details in Supporting information, 2.3 Preparation of lipase‐polymer conjugates) via the three‐step method: i) carbodiimide (EDC)‐mediated cationization of solvent‐accessible carboxylates to produce a fully multi‐cationic protein (c8M or cMb), or succinylation of solvent‐exposed lysine residues to produce a protein with the predominance of surface negative charges (a8M or aMb). MALDI‐TOF mass spectra gave 100% of cationization efficiency (18 carboxylates modified) and 80% of anionization efficiency (8 amines modified) for 8 M (Figure S5A, Supporting Information); and 82% of cationization (17 carboxylates modified) and 100% of anionization (20 amines modified) efficiencies for Mb. Moreover, the TNBS assay confirmed the ∼90% succinylation efficiency, in accordance with MALDI‐TOF results (Figure S5B, Supporting Information), indicating effective modification of surface *α*‐amines by succinic anhydride. This was consistent with zeta potential measurements that showed a significant increase and a moderate decrease in surface charge from −21.1 ± 2.0 mV to 70.5 ± 7.6 and −27.1 ± 1.6 mV for the cationized and anionized 8 M, respectively. Mb also showed a significant increase and a moderate decrease in surface charge from ‐2.3 ± 0.1 mV to +65.4 ± 3.1 and −11 ± 1.6 mV after cationization and anionization, respectively. ii) electrostatic coupling of PEG‐based anionic and cationic surfactants to yield aqueous [c8M][S] (or [cMb][S]) and [a8M][cS] (or [aMb][cS]) conjugates, respectively, followed by thorough dialysis to remove excessive unbound surfactants. In the case of 8 M, electrostatic conjugation with anionic and cationic surfactants to produce aqueous dispersions of the corresponding ionic nanoconstructs were associated with a concomitant decrease and increase in the zeta potential to −5.2 and 6.0 mV, respectively. It was illustrated that [c8M][S] and [a8M][cS] conjugates displayed similar molar ratios of protein to bounded polymers, which were estimated with BCA method (Table S1, Supporting Information). On the other hand, [cMb][S] and [aMb][cS] exhibit the zeta potential of −25.4 ± 1.1 and 16.7 ± 1.3 mV, respectively, suggestive of more excessive bounded polymers for [cMb][S] and [aMb][cS] conjugates, which respectively displayed 108 and 138 polymers per protein molecule calculated with BCA method (Table S1, Supporting Information); and iii) lyophilization of the nanoconjugate solutions to afford freeze‐dried ionic nanoconstructs that subsequently underwent thermally induced melting as a transparent liquid at ≈60 °C. As is shown in **Figure** **2**, the cS polymer displayed a melting transition at 29.5 °C, which is much lower than the S polymer (38 °C). It seemed that the significant melting point depression observed in the cationic polymer was due to the enhanced miscibility via the introduction of two methyl groups in the hydrophilic head of surfactants.^[^ ^15^ ^]^ Accordingly, this may be why both solvent‐free liquid [a8M][cS] and [aMb][cS] exhibited lower melting points than their respective cation‐type protein liquids (Figure 2).

**Figure 3:**
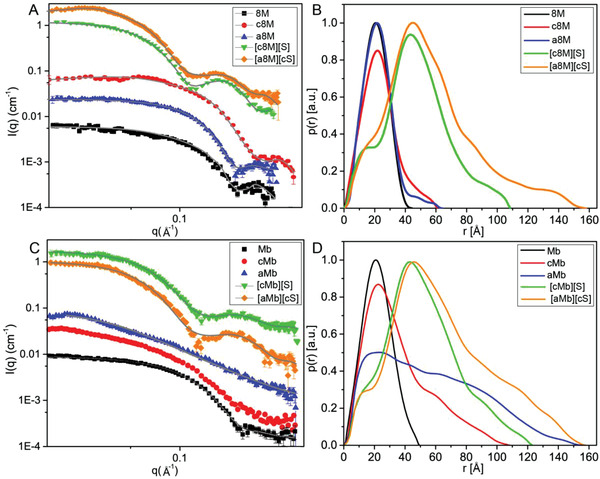
Small‐angle X‐ray scattering data (SAXS) of native and modified 8M A) and Mb C) in aqueous environment. Fits to data are shown as a solid line. The corresponding *p(r)* functions (a.u.) as a function of distance *r* (Å) obtained with the IFT procedure for native and modified 8M C) and Mb D) in aqueous condition. The native enzymes are buffered in 10 mM sodium phosphate buffer, pH 6.8 while cationized and polymer‐conjugated enzymes are all unbuffered for their high solubility in Milli‐Q water.

**Characterization of the synthesized biofluids**. Structural characterization was performed throughout all stages of biofluid synthesis with synchrotron radiation circular dichroism (SRCD), small‐angle X‐ray scattering (SAXS) and attenuated total reflection Fourier transform infrared (ATR‐FTIR). We first turned to SAXS to investigate the effects of anionization and cationization on 8M and Mb structures. Native 8M and Mb could both be fitted on absolute scale with their corresponding crystal structures (pdb:1ISP^[^ ^12^ ^]^ and 1MBN^[^ ^16^ ^]^) (**Figure** **3**A,C). We used the pair‐distance distribution function *p*(*r*) to get model‐independent information on the shapes and sizes of the proteins (Figure 3B,D and Table S2, Supporting Information). From the *p*(*r*) it is shown that anionization and cationization has little effect on 8 M while Mb has a markedly changed structure after both of the modification processes. The stability of 8M after modifications was further confirmed by fitting the crystal structure to the data and only a small increase in size could be seen going from *R_g_* = 1.6 ± 0.1 nm (8M) to *R_g_* = 1.8 ± 0.2 nm (a8M) and *R_g_* = 1.8 ± 0.1 nm (c8M). For a8M, the crystal structure fitted only slightly worse than for 8M, while a structure factor describing protein‐protein repulsion had to be included for c8M, which is likely related to the strong zeta potential seen for c8M compared to a8M that cause a stronger repulsion. cMb and aMb both showed highly elongated structures but were still in their monomeric form. aMb showed the most elongated structure (*R_g_* = 7.3 ± 0.1 nm) and could be fitted with a model describing a completely unfolded random coil protein. cMb (*R_g_* = 3.5 ± 0.1 nm) had a somewhat more compact structure than aMb but less compact than Mb (*R_g_* = 1.7 ± 0.1) and the data could not be fitted with a model describing neither a random coil structure or that from the crystal structure.

The anionic polymer surfactants (S) have been characterized with SAXS in previously reported work, in which they were called S_7_ instead.^[^ ^3^, ^13^ ^]^ The two polymers have a very similar structure and are only different in their headgroup and measurements of the polymers revealed highly similar sizes of the two polymers (Figure S1, Supporting Information). The data was fitted with a core‐shell model as seen earlier.^[^ ^13^ ^]^ One of the major differences between the two polymers was that while S showed inter‐micelle interactions cS showed strong inter‐micelle repulsions and this had to be included in the model to fit the data. Fitting also revealed similar aggregation numbers for the two polymers with S having an aggregation number of 37 and cS with an aggregation number of 38. This suggests that the difference in polymer headgroup mainly affected the inter‐micelle interaction while not affecting the overall structure to a large degree.

For polymer‐conjugated Mb and 8M samples, a clear “bump” was seen at high *q*‐values suggesting that the formed structures were core‐shell structures as seen before for other similar protein‐polymer conjugates.^[^ ^6^, ^13^ ^]^ The data was fitted by assuming that the core consisted of folded protein while the shell consisted of the polymer. Fitting was done on an absolute scale and to reduce fitting parameters, the core dimensions was kept constant to that of Mb (*r* = 24.8 Å and eccentricity *ε* = 0.56) or 8M (*r* = 23.2 Å and *ε* = 0.72). The model fitted well in the case of [a8M][cS] and [c8M][S] while [aMb][cS] and to some extent [cMb][S] both had issues in fitting the minima seen in the data. This suggests that the core‐size of the protein‐conjugates were different from that of folded proteins. Fitting was worse for [aMb][cS] suggesting that especially aMb was not completely refolded upon conjugation. This was also the case for [cMb][S], even though not as pronounced. Therefore, even though cMb and aMb regained some of its native structure when conjugated, they did not completely refold.

**Figure 4:**
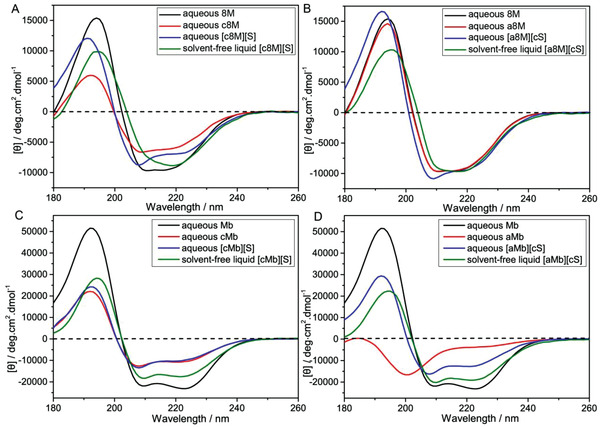
SRCD spectra of aqueous and solvent‐free liquid samples during synthesis of biofluids [c8M][S] A), [a8M][S] B), [cMb][S] C) and [aMb][cS] D). SRCD spectra of aqueous and solvent‐free liquid samples during synthesis were measured at 25 and 45 ^o^C, respectively.

The secondary structure was further investigated using SRCD (**Figure** **4**), which well supported the structural information yielded by SAXS. 8M underwent a loss of ellipticity after cationization under ambient conditions (Figure 4A) while anionized 8M showed similar spectra to the native enzyme (Figure 4B). Deconvolution of the CD spectra showed that surface cationization resulted in a significant decrease in *α*‐helix associated with an increase of *β*‐sheet and of unordered domains for c8M, when compared with native 8M, while a8M displayed negligible change in secondary structure distribution after anionization (**Table** **1**). Cationization can disrupt all surface salt bridges that can help maintain 8M globular architecture, while succinylation of 8M left four surface arginine residues unreacted and involved in the existing salt bridges. Polymer conjugation helped in recovering the secondary structure of the cationized enzyme to some extent as reported before^[^ ^6^ ^]^ (Figure 4A), whereas the recovered ellipticity of the polymer‐conjugated c8M was still lower than that of [a8M][cS] (Figure 4B). The finding indicated that the surface charge modification plays a significant role in influencing enzyme secondary structure with the neutralization of surface charges by conjugating polymer surfactants.

**Table 1:** Secondary structure content in various 8M and Mb constructs

Solvent	Samples	*α*‐Helix/%	*β*‐Sheet/%	Turns/%	Unordered/%	NRMSD^c)^
10 mM sodium phosphate, pH 6.8^a)^	8M	24	30	11	35	0.033
	c8M	13	36	12	39	0.05
	a8M	23	30	12	35	0.034
	[c8M][S]	18	30	14	38	0.044
	a8M][cS]	24	27	13	36	0.03
	Mb	70	4	9	17	0.004
	cMb	36	18	14	32	0.019
	aMb	7	31	16	46	0.037
	[cMb][S]	38	17	14	31	0.023
	[aMb][cS]	45	14	14	27	0.014
Solvent‐free liquid^b)^	[c8M][S]	18	34	12	36	0.024
	[a8M][cS]	24	27	13	36	0.05
	[cMb][S]	55	7	12	26	0.018
	[aMb][cS]	58	8	12	22	0.011

^a)^ Estimated secondary structure contents of the aqueous samples at 25 °C evaluated by deconvolution of synchrotron radiation circular dichroism spectra.

^b)^ Estimated secondary structure contents of the biofluids at 45 °C evaluated by deconvolution of synchrotron radiation circular dichroism spectra.

^c)^ NRMSD is the abbreviation of Normalized Root‐Mean‐Square Deviation.

**Figure 5:**
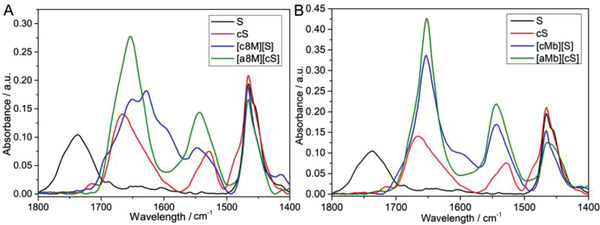
ATR‐FTIR spectra showing amide regions for solvent‐free liquid [c8M][S] (blue) and [a8M][cS] (green). The spectra of anionic surfactants (black) and cationic surfactants (red) are also shown.

Deconvolution of the SRCD spectrum of solvent‐free liquid [a8M][cS] indicated a similar secondary structure distribution with its aqueous precursors (Table 1) in spite of the reduction in the intensity of the characteristic maxima at 192 nm after conversion of the physical state from aqueous solution to solvent‐free liquid phase (Figure 4). Significantly, [a8M][cS] biofluid displayed higher active *α*‐helix content than biofluid [c8M][S] as is in anhydrous environment (Table 1), further supported by ATR‐FTIR spectra, in which [a8M][cS] displayed typical Amide I and Amide II peaks at ≈1655 and 1540 cm^–1^, respectively, while [c8M][S] exhibited an extra Amide I peak at ≈1633 cm^–1^ that suggested higher *β*‐sheet content (**Figure** **5** **A**). The advantage of anion‐type biofluid in terms of structure retention in the case of 8M was further supported by activity assays showing that 8M retained majority of its activity during synthesis of anion‐type biofluid rather than the cation‐type one and the activity of ultimate solvent‐free liquid [a8M][cS] is 3.5‐fold over its cation‐type counterpart (**Table** **2**). As stated above, the introduction of methyl groups in the end of the polymer surfactants may enhance polymer miscibility,^[^ ^15^ ^]^ which might further improve the miscibility of the resultant protein liquids, thus leading to higher structure retention and enzyme activity.

**Table 2:** Enzyme activities^a)^ of the native, cationized and polymer‐conjugated lipase variants in aqueous solutions and solvent‐free liquids

	Enzyme activity µmolmin^‐1^mg^‐1^		Enzyme activity µmolmin^‐1^mg^‐1^
Solvent‐free liquid [c8M][S]	2.6 ± 0.5	Solvent‐free liquid [a8M][cS]	9.2 ± 1.3
Aqueous [c8M][S]	0.6 ± 0.1	Aqueous [c8M][S]	6.0 ± 0.9
Aqueous c8M	2.1 ± 0.2	Aqueous a8M	7.9 ± 0.7
Aqueous 8M	9.1 ± 0.5	Aqueous 8M	9.1 ± 0.5

^a)^ Enzyme activities of the solvent‐free liquid lipases at 50 °C and the aqueous precursors at 25 °C.

The presented data are the means ± standard deviation from three separate determinations (*p* < 0.05).

**Figure 6:**
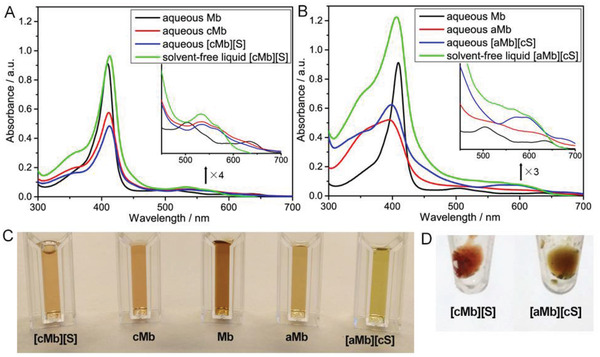
A) UV‐vis spectra of native and modified Mb in synthesis of solvent‐free liquid [cMb][S]; B) UV‐vis spectra of native and modified Mb in synthesis of solvent‐free liquid [aMb][cS]. The aqueous and solvent‐free liquid samples are measured at 25 and 45 ^o^C, respectively. C) Visual appearance of native and modified myoglobin in aqueous environment; D) visual appearance of solvent‐free liquid [cMb][S] and [aMb][cS].

In the case of Mb, cationization resulted in partial unfolding of Mb as is described before (Figure 4C and Table 1)^[^ ^2^ ^]^ while anionization lead to complete unfolding, which is reflected by SRCD spectra showing the absence of characteristic minima at 222 nm and 208 nm after anionization of Mb (Figure 4D). Significantly, deconvolution of the CD spectra showed that the level of *α*‐helical secondary structure in the aqueous aMb molecule was 7% compared with 70% in native Mb and 36% in cMb, and that this was associated with a significant increase in the content of the *β*‐sheet, turn and unordered domains from 4, 9 and 17% to 31, 16 and 46%, respectively (Table 1). The pronounced impacts of both increasing the surface positive and negative charges on Mb secondary structures may be consistent with the absence of disulfide bridging and thereby strong dependence of Mb globular structure on inter‐ and intra‐ helix electrostatic stabilization as reported before.^[^ ^2^, ^17^, ^18^ ^]^ However, the much more marked influence of anionization than that of cationization upon Mb secondary structure seemed more complex to explain. Interestingly, the heme group did not separate from the peptide and displayed a blue‐shifted broad Soret band in aMb compared with the red‐shifted Soret band observed in the case of cMb (**Figure** **6**A,B). Strikingly, conjugation of cationic polymer surfactants resulted in partial recovery of aMb secondary structure with *α*‐helical content increasing up to 45%, which was even higher than that of [cMb][S]. This is in accordance with the SAXS data that showed a globular structure even though complete refolding was not achieved. In addition, [cMb][S] conjugates displayed a further red‐shifted Soret band as is similarly illustrated in a previously reported study.^[^ ^2^ ^]^ Similarly, conjugation of cationic polymer surfactants resulted in red‐shift of the Soret band of aMb, but still blue‐shifted compared with that of native Mb. Interestingly, [aMb][cS] solutions turned out green while the [cMb][S] solution is red‐brown (Figure 6C). In general, the color of myoglobin could be changed to green via reactions between myoglobin and hydrogen sulfide (H_2_S), highly oxidizing agents (i.e., hydrogen peroxide), or excess nitrite (or HNO_2_) under specific conditions to form sulphmyoglobin, choleglobin or nitrimyoglobin.^[^ ^19^, ^20^, ^21^, ^22^ ^]^ The study on the mechanism underlying green discolouration of myoglobin induced by atmospheric pressure plasma (APP) indicated that nitrites generated by APP can react with myoglobin to exclusively form nitrimyoglobin which has the green color, which was associated with a decrease in the absorption peaks at 503 and 630 nm with an increase in the peak at 590 nm in UV absorption spectrum of APP‐treated myoglobin solution.^[^ ^23^ ^]^ In the present study, the UV spectrum of [aMb][cS] solution also revealed a new broad peak at 590 nm (Figure 6B), indicating that the green discolouration of aMb after conjugation of cationic polymers might be resulted from the formation of nitrimyoglobin,^[^ ^23^ ^]^ which is worth further exploration.

UV‐vis spectra of the solvent‐free [cMb][S] showed Soret band at 413 nm, which was further red‐shifted when compared with its aqueous precursor (Soret = 412 nm) (Figure 6A). Conversely, the [aMb][cS] conjugate showed a red‐shifted Soret band (Soret = 405 nm) when compared with aqueous [aMb][cS] conjugates after transformation of the physical state from aqueous solution to solvent‐free liquids via lyophilization and thermal annealing, whereas its Soret band was still red‐shifted when compared with aqueous native Mb (Figure 6B). Meanwhile, [aMb][cS] biofluids still displayed green discoloration (Figure 6D), associated with the retention of a relatively high shoulder peak in its UV‐vis spectrum (Figure 6B). Structurally, solvent‐free liquid [aMb][cS] and [cMb][S] displayed similar secondary structure distribution, in which the *α*‐helical contents of solvent‐free liquid [cMb][S] and [aMb][cS] were increased to 55%–58% (Table 1). This was confirmed by ATR‐FTIR spectroscopy, which showed strong amide I and II bands at 1655 and 1540 cm^−1^, respectively, typical for relatively high *α*‐helical levels for solvent‐free liquid [cMb][S] and [aMb][cS] (Figure 5B). The findings indicated that the natively partially folded anion‐type [aMb][cS] biofluids could be achieved in spite of the complete unfolding resulting from anionization, suggestive of a certain applicability for anion‐type biofluids.

**Figure 7:**
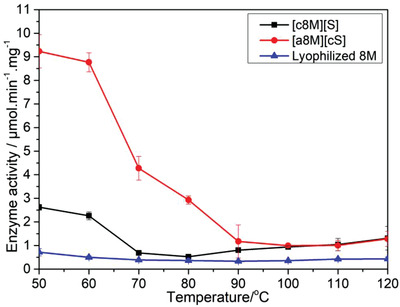
Temperature‐dependent activity assay for solvent‐free liquid [c8M][S] (black line), solvent‐free liquid [a8M][cS] (red line) and lyophilized 8M (blue line). The presented data are the means ± standard deviation from three separate determinations (*p* < 0.05).

**Thermostability of the synthesized biofluid lipases**. To compare the thermostability of solvent‐free liquid [c8M][S], [a8M][cS] as well as lyophilized 8M, we monitored the temperature dependence of the rate of p‐nitrophenol (pNP) formation from hydrolysis of p‐nitrophenyl butyrate (pNPB) catalyzed by solvent‐free liquid lipases (**Figure** **7**). Although [a8M][cS] liquids showed higher activity than [c8M][S] liquids at operating temperatures ranging from 50 to 100°C, [a8M][cS] underwent distinct decrease in catalytic reaction rates with increasing temperature, and the ascendancy of [a8M][cS] activity over [c8M][S] gradually diminished to zero at 100°C, and [c8M][S] and [a8M][cS] biofluids showed similar activity, which slowly elevated as temperature increased up to 120 °C (Figure 7). Results indicated that it might be difficult for the solvent‐exposed catalytic site to maintain high active conformation at relatively higher temperatures even in solvent‐free liquid form. Meanwhile, both of c8M][S] and [a8M][cS] biofluids displayed higher activities than lyophilized 8M at 50–120 °C, indicative of the significance of the existence of the surrounding polymer surfactants that could not only help maintain native enzyme structures but also function as solvents for diffusion of the substrates and the hydrolytic products.^[^ ^3^ ^]^

Solvent‐free liquid [c8M][S] and [a8M][cS] biofluids were further characterized in terms of structural stability with SRCD (Figure S2, Supporting Information). As reported before,^[^ ^6^ ^]^ [c8M][S] liquids underwent a reduction in the intensity of characteristic minima at 222 nm when the temperature increased from 42.7 to 84.5 °C, associated with the *α*‐helix content decreasing from 23 to 19% (Figure S2C, Supporting Information). On the contrary, the characteristic minima of [a8M][cS] CD spectra at 222 nm remained almost unaltered during heating while a decrease in the intensity of the characteristic minima at 210 nm occurred at the same time (Figure S2B, Supporting Information). The deconvoluted data revealed that the *α*‐helical content of [a8M][cS] remained stable as the temperature increased (Figure S2D, Supporting Information). This indicates that the thermostability of [a8M][cS] liquids is higher than [c8M][S], and the significant loss in catalytic activity of [a8M][cS] liquids upon heat treatment might be attributed to the heat‐induced perturbation of the active site. Nevertheless, [a8M][cS] still displayed higher activities than [c8M][S] in protein liquids ranging from 50 to 100 °C, and both liquids showed moderate improvement in activity at temperatures over 100°C (Figure 7), probably due to contributions from the increase in kT (Arrhenius behavior), as well as from temperature‐induced reduction of viscosity that leads to increased substrate and product diffusion rates.^[^ ^3^, ^6^ ^]^

## 3 Discussion

In summary, we have developed a new class of anion‐type protein liquids ([a8M][cS] and [aMb][cS]) via anionization and subsequent conjugation of cationic polymer surfactants based on established theory.^[^ ^1^, ^4^, ^5^ ^]^ For proteins rich in surface carboxylates, succinylation of protein surface amines (Lysine side chains and N‐terminal *α*‐amine) may not only result in maintained enzyme structure and activity (a8M) but also result in complete unfolding (Mb). Strikingly, conjugation of cationic polymer surfactants could further maintain a8M structure and activity as well as lead to partially refolding of aMb in both aqueous conjugates and protein liquids, accompanied by alterations in optical activity. In protein liquids, [a8M][cS] showed higher activity and thermostability than [c8M][S], and [aMb][cS] retained higher *α*‐helical level than its cation‐type counterpart. This may be due to the enhanced miscibility of the biofluids that the conjugated cationic polymers contribute to. Together, these results suggest that the anion‐type biofluid enzymes can better overcome the negative impact polymer conjugation might cause in terms of loss of activity and might therefore exhibit application potential, which is well worth further exploration.

## 4 Experimental Section

### Synchrotron Radiation Circular Dichroism (SRCD) Spectroscopy

Far‐UV SRCD spectra of aqueous solutions of native, cationized/anionized and polymer‐conjugated proteins (8M and Mb) were recorded between 280 and 170 nm using AU−CD beamline on the ASTRID2 storage ring (ISA, Aarhus University, Denmark). A quartz cuvette with 0.1 mm of path length was used and protein concentrations (typically 1 mg ml^−1^ buffered in 10 mM sodium phosphate, pH 6.8) were all determined by BCA method.

Millidegree ellipticity was converted to mean residue molar ellipticity (*θ*, deg cm^2^ dmol^–1^) using Equation (1). Ellipticity is the millidegree ellipticity in machine units (mdeg); *L* is light path length in centimeters (cm); *c* is the protein concentration in micromolar (µM); and *n* is the number of peptide bonds in the protein and ellipticity is the raw data from the instrument.

(1) $$\theta =\frac{\mathrm{Ellipticity}\times 10^{7}}{L\cdot c\cdot n}$$

SRCD spectra of polymer‐based solvent‐free liquid proteins were collected over the wavelength range 170–280 nm with an integration time of 2 s per point and 1 nm data interval. Samples were cast as thin films between two synthetic quartz plates, which were incubated at 50 °C on a heat block. The samples were incubated at each temperature for 5 min prior to CD spectrum scan.

The UV‐vis absorbance spectra of the samples were measured simultaneously with the CD spectra. According to the Beer–Lambert law (Equation (2)), the absorbance (A) is proportionally correlated with the sample molar concentration (c (molar units, M)), the light path length in centimeters (L, cm), and the molar absorptivity/molar extinction coefficient (*ε*, M^–1^ cm^–1^) for the sample at the specified wavelength (*λ* = 205 nm).^[^ ^24^, ^25^ ^]^

(2) $$A_{205}=\varepsilon_{205}\cdot c\cdot L$$

As shown in Figure S6, Supporting Information, the anionic surfactants showed almost no absorbance at 205 nm while the cationic ones had much higher A_205_ values, which could be due to the reacted 3‐dimethylaminopropylamine and the formed amide bonds. Hence a8M (or aMb) as well as the coupled cationic surfactants contributes to the UV absorbance (Equation (3)).

(3) $$A_{205}\left(\mathrm{total}\right)=\varepsilon_{205}\left(a\mathrm{Protein}\right)\cdot c\left(a\mathrm{Protein}\right)\cdot L+\varepsilon_{205}\left(cS\right)\cdot c\left(cS\right)\cdot L$$

The molar extinction coefficient at 205 (*ε*_205_) nm for either cationized or anionized protein (8 M or Mb) is regarded as being similar to the native one, of which the *ε*_205_ is calculated based on the amino acid sequence and the amino acids which have a measurable extinction coefficient at this wavelength.^[^ ^26^ ^]^ This can be calculated using an online server,^[^ ^26^ ^]^ and the results turned out to be 666340 and 607 090 M^‐1^ cm^–1^ for 8 M and Mb, respectively. In order to estimate *ε*_205_ value of the cationic surfactant, the melted surfactants were cast in a two‐part quartz cell (0.01 mm path length), followed by the SRCD spectra scan that could deliver the A_205_ value of the sample. The molar concentration of the melt surfactants was estimated to be ≈2740 M^–1^ cm^–1^ by the molecular weight and density of the surfactants as 1326 g mol^–1^ and 1.1 g cm^–3^, respectively. Furthermore, molar ratio of [aProtein] ([a8M] or [aMb]) to [cS] can be estimated by both BCA method and SAXS measurements. Accordingly, the indeed UV absorbance of the protein in [aProtein][cS] liquids could be estimated by Equation (4):

(4) $$A_{205}\left(a\mathrm{Protein}\right)=\frac{A_{205}\left(\mathrm{total}\right)\cdot \varepsilon_{205}\left(a\mathrm{Protein}\right)\cdot c\left(a\mathrm{Protein}\right)}{\varepsilon_{205}\left(a\mathrm{Protein}\right)\cdot c\left(a\mathrm{Protein}\right)+\varepsilon_{205}\left(cS\right)\cdot c\left(cS\right)}$$

According to Equation (2), L·c (cm·µM) required for calculation of the mean residue molar ellipticity in Equation (1) can be obtained with Equation (5):

(5) $$L\cdot c=\frac{A_{205}\times 10^{8}}{\varepsilon_{205}}$$

Hence the mean residue molar ellipticity (*θ*, degcm^2^ dmol^–1^) data of solvent‐free liquid samples can be calculated using:

(6) $$\theta =\frac{\mathrm{Ellipticity}\cdot \varepsilon_{205}}{A_{205}\cdot 10n}$$

Secondary structure content estimation was performed using the DICHROWEB service with the CDSSTR algorithm and SP175 data set.^[^ ^27^, ^28^, ^29^ ^]^ Spectra were fitted between 175 and 240 nm, with resultant fits only accepted when the normalized root mean squared deviations (NRMSDs) were below 0.05.

### Activity Measurements

All enzyme specific activities were determined at 25 °C by measurements performed on Shimadzu UV‐2700 spectrophotometer with a water bath temperature control device using p‐nitrophenyl butyrate (pNPB) as the substrate. Briefly, 780 µl of 50 mM sodium phosphate buffer (pH 7.2) was incubated at a desired temperature for 3 min for temperature equilibration in 1‐cm cuvette within the cuvette holder to which 2–8 µg of biocatalysts (native, cationized/anionized and polymer‐conjugated lipases) was added and incubated further for 1 min. Reaction was started by adding 10 µl of pNPB from 80×stock (40 mM pNPB dissolved in acetonitrile), and the rate of increase in absorbance at 410 nm was recorded. The rate of spontaneous hydrolysis was also monitored for correction. Protein quantitation of all samples was performed by the BCA method.^[^ ^30^ ^]^

The activities of all freeze‐dried enzyme‐polymer conjugates was measured by monitoring the average rate of enzyme‐catalyzed hydrolysis of anhydrous liquid pNPB within the first 30 seconds. All enzyme‐polymer conjugate solutions (pH 8.0) were lyophilized for 48 h from the aliquots of solutions that each contain 0.05 mg of proteins, which were distributed into eppendorf tubes according to BCA Protein Assay results. Each sample‐containing tube was incubated at various temperatures (50−120 °C) for 5 min, followed by addition of 0.2 µl of desiccated pNPB. The tube was immediately closed and incubated at the respective temperature for 0.5 min, and then 1 ml of quenching buffer (50 mM sodium phosphate, 0.5% SDS, pH 7.2) was added into the tube in order to resolve all reaction mixtures as well as quench the reaction by denaturing enzymes. SDS has been proved to be highly effective in inhibiting the residual activities of lipases in the colorimetric assay,^[^ ^31^ ^]^ and it was also experimentally proved to effectively inhibit the enzyme activity in the presence of conjugated polymers. The amounts of products were determined by assaying the absorbance at 412 nm at room temperature, and the hydrolysis of 0.2 µl of pNPB itself at various temperatures were also assayed as the control. Each of the above activity assays were performed in triplicate, and the data were expressed as “mean value ± SD” (standard deviation, *n* = 3).

### Statistical Method

Data processing was performed in Microsoft Excel 2013 (Microsoft Corp. Redmond, WA, USA). All measurements were conducted in triplicates, and the results are reported as means ± standard deviations. One‐way analysis of variance (one‐way ANOVA) was performed using Microsoft Excel Analysis Toolpak (2010) to identify significant differences between groups (*p* < 0.05). All figures were drawn by Origin pro 2016 software (Origin lab Corp. Northampton, MA, USA) and Adobe Illustrator CC 2013 (Adobe Systems Incorporated, San Jose, CA, USA).

## Conflict of Interest

The authors declare no conflict of interest.

## Supporting information

Supporting Information

## Data Availability

The data that support the findings of this study are available from the corresponding author upon reasonable request.
