# Supplementary material for: Superanionic Solvent‐Free Liquid Enzymes Exhibit Enhanced Structures and Activities
Source: Adv Sci (Weinh). 2022 Aug 21;9(32):2202359. doi: 10.1002/advs.202202359 (PMC9661855; doi:10.1002/advs.202202359)
Supplement: Supplementary file 1 — Supporting Information [file ADVS-9-2202359-s001.pdf]

ADVANCED SCIENCE
Open Access

Supporting Information

for *Adv. Sci.*, DOI 10.1002/advs.202202359

Superanionic Solvent-Free Liquid Enzymes Exhibit Enhanced Structures and Activities

Ye Zhou, Jannik Nedergaard Pedersen, Jacob Nedergaard Pedersen, Nykola C. Jones, Søren Vrønning Hoffmann, Steen Vang Petersen, Jan Skov Pedersen, Adam Perriman, Renjun Gao and Zheng Guo**

Supporting Information

Superanionic solvent-free liquid enzymes exhibit enhanced structures and activities

Ye Zhou[a,b], Jannik Nedergaard Pedersen[c], Jacob Nedergaard Pedersen[b], Nykola C. Jones[d], Søren Vrønning Hoffmann[d], Steen Vang Petersen[e], Jan Skov Pedersen[c], Adam Perriman[f], Renjun Gao*[a], Zheng Guo*[b]

[a] Key Laboratory for Molecular Enzymology and Engineering, the Ministry of Education, School of Life Sciences, Jilin University, No. 2699, Qianjin Street, Changchun 130012 (China)
[b] Department of Engineering, Aarhus University, Gustav Wieds Vej 10, Aarhus 8000 (Denmark)
[c] Department of Chemistry and Interdisciplinary Nanoscience Center (iNANO), Aarhus University, Gustav Wieds Vej 14, Aarhus 8000 (Denmark)
[d] ISA, Department of Physics and Astronomy, Aarhus University, Ny Munkegade 120, Aarhus 8000 (Denmark)
[e] Department of Biomedicine, Aarhus University, Wilhelm Meyers Allé 4, Aarhus 8000 (Denmark)
[f] School of Cellular and Molecular Medicine, University of Bristol, Bristol BS8 1TS (UK)
**To whom correspondence should be addressed*
Zheng Guo; email: guo@eng.au.dk ; Renjun Gao; email: gaorj@jlu.edu.cn

Supplementary Tables and Figures

Table S1. MALDI-TOF MS and BCA method results

	[c8M][S]	[a8M][cS]	[cMb][S]	[aMb][cS]
Theoretical molecular weight (Mw)	19600.9	19600.9	17082.7	17082.7
Mw of native proteins (MALDI-TOF)	19631.1	19631.1	16960.6	16960.6
Mw of cationized proteins (MALDI-TOF)	21084.9	-	18406.2	-
Increased protein Mw resulted from cationization	1453.8	-	1445.6	-
Number of cationized sites[a]	17.3	-	17.2	-
Binding sites towards anionic polymer surfactants	33	-	38	-
Molar ratio of protein to polymer in cation-type biofluids (BCA assay)[b]	70 ± 7	-	108 ± 3	-
Mw of anionized proteins (MALDI-TOF)	-	20438.4	-	19060.7
Increased protein Mw resulted from anionization	-	837.5	-	2100.1
Number of anionized sites [c]	-	8.4	-	21
Binding sites towards cationic polymer surfactants	-	27	-	42
Molar ratio of protein to polymer in cation-type biofluids (BCA assay)[b]	-	72 ± 5	-	138 ± 6

[a]Theoretically, cationization of each site would lead to an increase in protein molecular weight by 84.18 g/mol, which was used for calculation of cationized sites.

[b]The molar ratio of protein to polymer surfactant was calculated by resolving a certain amount of free-dried conjugates (with known mass) into deionized water, followed by protein quantitation with BCA method.

[c]Theoretically, anionization of each site would lead to an increase in protein molecular weight by 100 g/mol, which was used for calculation of cationized sites.

Table S2. Fitting parameters from the IFT procedure based on SAXS data.

sample	radius of gyration (R_g, nm)	maximum dimension (D_{max}, nm)
8M	1.6 ± 0.1	4.5
c8M	1.8 ± 0.1	6.0
a8M	1.8 ± 0.2	6.2
[c8M][S]	3.9 ± 0.2	12
[a8M][cS]	4.3 ± 0.3	16.0
Mb	1.7 ± 0.1	5.0
cMb	3.5 ± 0.1	11.0
aMb	7.3 ± 0.1	15.5
[cMb][S]	4.0 ± 0.1	12.5
[aMb][cS]	5.1 ± 0.1	16.0

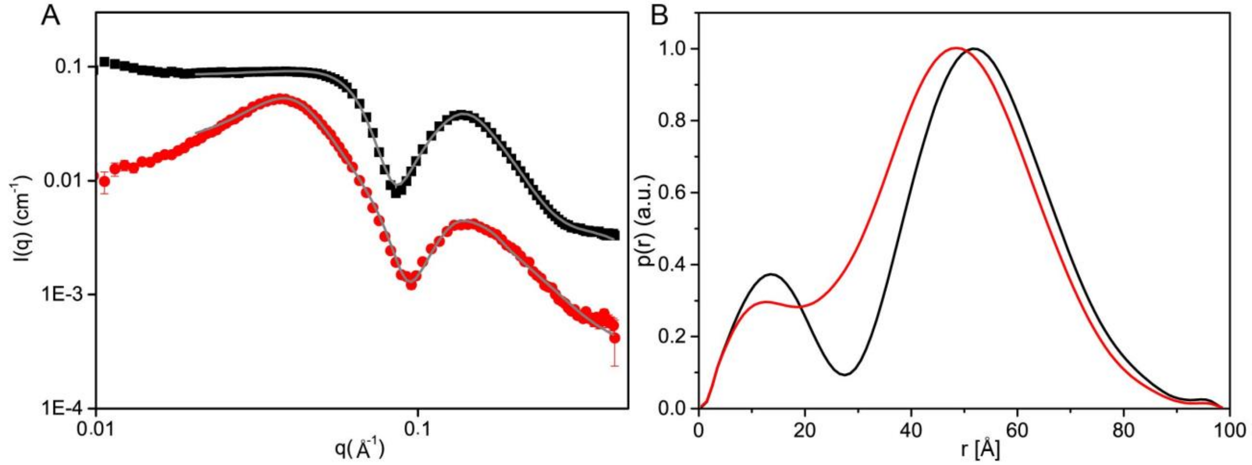

Figure S1. (A) Small-angle X-ray scattering data (SAXS) of anionic (black squares) and cationic (red circles) polymer surfactants in Milli-Q water. The fitted curves are shown in dark grey. (B) The corresponding *p(r)* functions (a.u.) as a function of distance *r*[Å] obtained with the IFT procedure for anionic (black curve) and cationic (red curve) polymer surfactants in Milli-Q water.

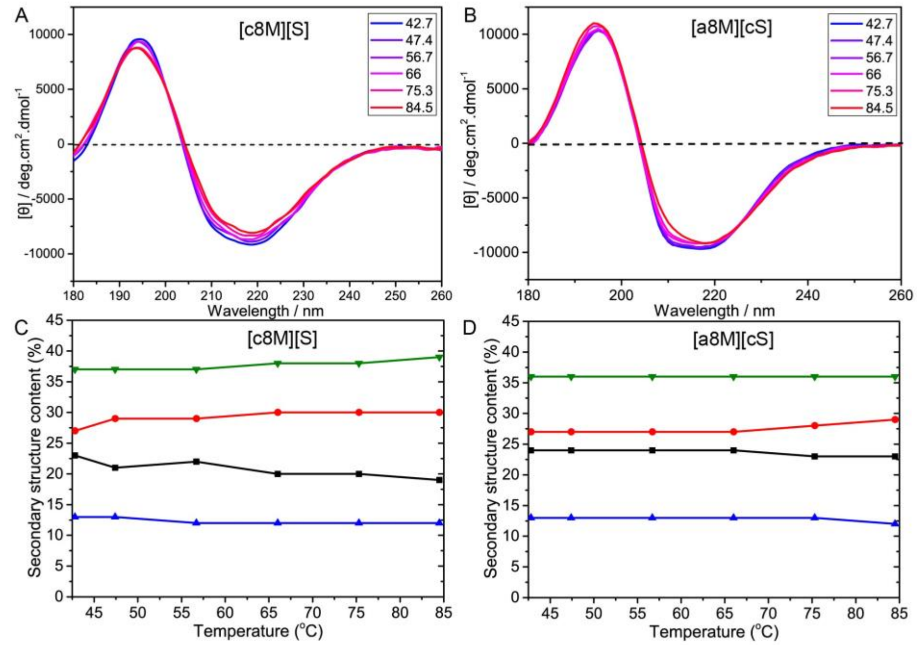

Figure S2. SRCD spectra of solvent-free liquid [c8M][S] (A) and [a8M][cS] (B) as a function of temperature (42.7-84.5 ^{o}C), and the deconvoluted temperature-dependent changes in the distribution of α-helices (black squares), β-sheets (red circles), turns (blue up-pointing triangles), and unordered domains (green down-pointing triangles) in solvent-free liquid [c8M][S] (C) and [a8M][cS] (D).

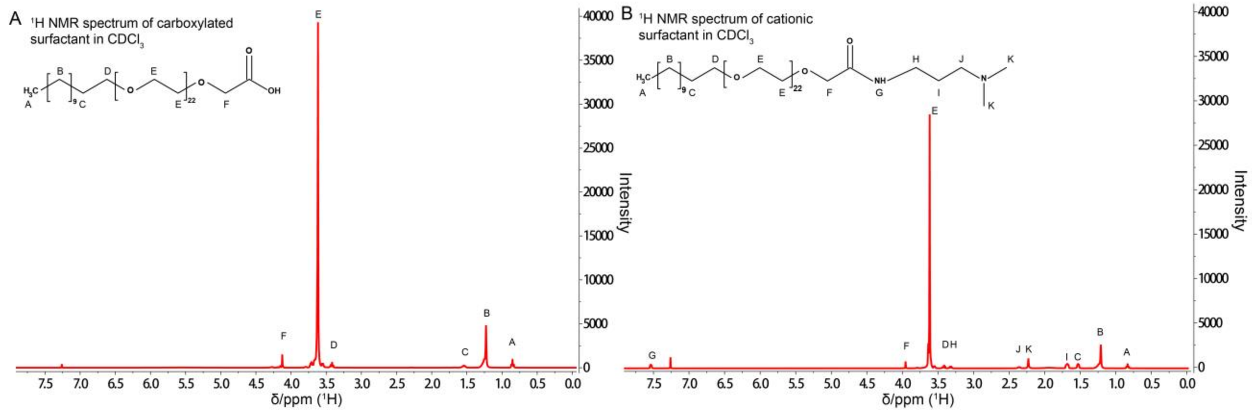

Figure S3: ^{1}H NMR spectrum of carboxylated polyoxyethylene (23) lauryl ether (A) and the derived cationic polymer (B) showing effective condensation with 3-dimethylaminopropylamine (DMAPA) as evidenced by the formation of five peaks corresponding to the protons on the reacted DMAPA.

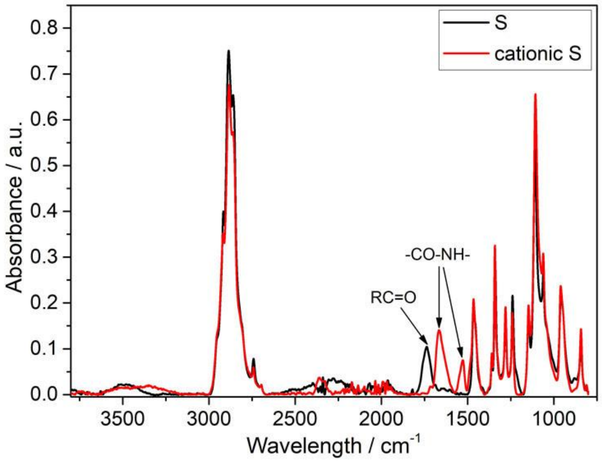

Figure S4: FTIR spectra of carboxylated polyoxyethylene (23) lauryl ether (black) and subsequently synthesized cationic polymer surfactant (red) showing the appearance of two Amide peaks at approximately 1668 and 1530 cm^{-1} after condensation, respectively.

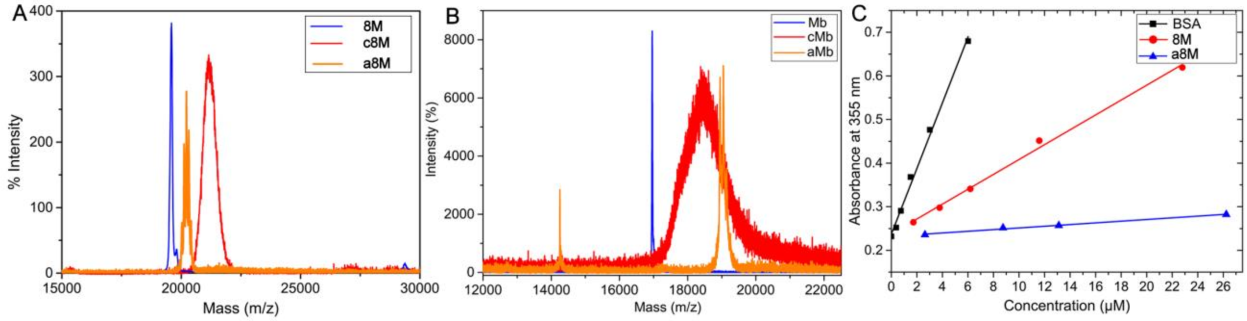

Figure S5: (A) MALDI-TOF mass spectrometry traces showing the effects of cationization (red) and anionization (orange) on the mass of 8M (blue). (B) TNBS assay for free amino groups. The absorbance values of TNBS-reacted BSA standard (black), native 8M (red) and succinylated 8M (blue) at 335 nm are plotted as a function of protein concentration.

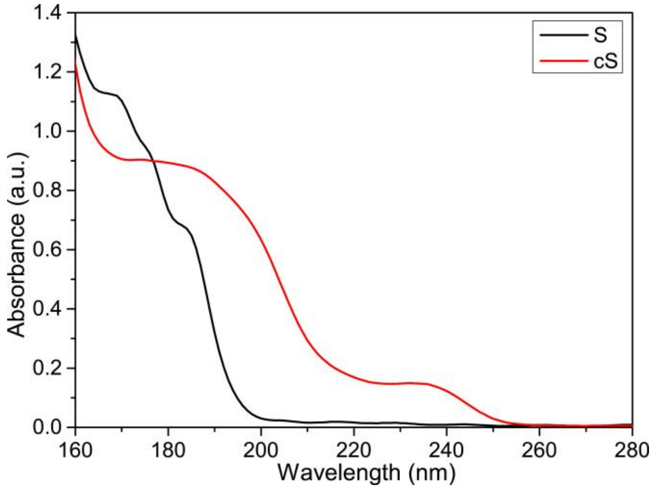

Figure S6: The Vis–Far UV (Vis–FUV) spectra of pure anionic (black line) and cationic (red line) polymer surfactants in solvent-free liquid form.

Supplementary Methods

1. Materials

Bacillus subtilis strain 168 was obtained from American Type Culture Collection (ATCC) and cultivated in Luria-broth (LB) medium. The genomic DNA was prepared by AxyPrepTM Bacterial Genomic DNA Miniprep Kit (Axygen, USA). *Escherichia coli* strains and plasmid pET-25b were from Novagen (Madison, WI, USA). Equine skeletal muscle myoglobin (Mb) was purchased from Sigma-Aldrich and used as received. All other chemicals, including N,N-dimethyl-1,3-propanediamine (DMPA), N-(3-dimethylaminopropyl)-N'-ethylcarbodiimide hydrochloride (EDC), 3-(dimethylamino)-1-propylamine (DMAPA), 3-dimethylaminopropylamine, 4-(4,6-dimethoxy-1,3,5-triazin-2-yl)-4-methylmorpholinium chloride (DMT-MM) and polyoxyethylene (23) lauryl ether (Brij L23) and succinic anhydride, were purchased from Sigma-Aldrich (St. Louis, MO, USA).

2. Methods

Synthesis of anionic and cationic polymer surfactants

The anionic polymer surfactants were synthesized via tempo-mediated oxidation of the terminal hydroxyl group of polyoxyethylene lauryl ether (Brij L23, Sigma-Aldrich)[1,2]. Briefly, 2 g of Brij L23 was dissolved in deionized water (50 ml) with NaBr (516 mg), TEMPO (52 mg) and NaClO (5 ml, available chlorine 10-15%). The solution was adjusted to pH 11 with 10 M sodium hydroxide and maintained while stirring for 24 h. The oxidation was quenched by the addition of 10 ml of ethanol, followed by continues stirring for 3 h. The reaction system was acidified to pH 1 with HCl, followed by extraction with 3×80 ml aliquots of chloroform. The solvent was then evaporated under reduced vacuum. The resulting pale yellow oil was redissolved in 50 ml hot ethanol followed by precipitation in the freezer overnight. The supernatant ethanol was removed by centrifuged at 4 °C followed by a second recrystallization with ethanol and evaporation under vacuum. The resultant colourless waxy solid was resolved in EtOH/Water (1:4 v/v) and dialyzed against deionized water for 24 h using Pur-A-Lyzer^{TM} Mega Dialysis Kit (1000 Da MWCO, Sigma-Aldrich), followed by the removal of water via lyophilization.

The cationic polymer surfactants were synthesized from the carboxylated Brij L23. Briefly, 5 g

of anionic surfactants (4 mmol) was resolved in 30 ml of acetone followed by addition of 3-dimethylaminopropylamine (748 μL, 6 mmol) and condensation reagent DMT-MM (1.66 g, 6 mmol). The mixture was stirred in a dark place for 3 hours at room temperature, followed by filtration and purification by chromatography (neutralized silica with MeOH/$CHCl_3$ (1:1 v/v) solvent). The resultant products were further evaporated, resolved in EtOH/Water (1:4 v/v) and dialyzed against deionized water for 24 h using the same low molecular weight cut-off membrane (1000 Da), followed by the removal of water via lyophilization. The purity of the products was determined with thin-layer chromatography and ^{1}H NMR spectroscopy. ^{1}H NMR showed effective oxidation with the formation of a peak at 4.16 ppm that corresponded to the protons on the α-carbon to the carboxylate (**Figure S3**), and effective condensation with the formation of a peak at 7.53 ppm that corresponded to the proton on the nitrogen that is involved in formed amide bond (**Figure S3B**).

Enzyme Cloning, Expression and Mutagenesis

The octuple mutant (8M) of *B. subtilis* lipase A was prepared as described before[3]. Briefly, the wild-type BsLA gene was cloned from the extracted genome DNA using a pair of primers as follows: forward primer 5'- GGAATTC<u>CATATG</u>GCAGAACACAATCCAGT-3' and reverse primer 5'- ATACCG<u>CTCGAG</u>TTAATTCGTATTCTGG-3', in which the restriction sites of Nde I and BamH I are underlined, respectively. The forward primer also introduced a start codon ATG, which encoded an extra methionine residue before the N-terminal alanine of mature protein, upon overexpression in *E. coli*. Codon optimization analysis and mutation experiments were performed on BsLA gene in order to improve expression level. For site-directed mutagenesis, BsLA was mutated to 8M (F17E, A20E, K44E, Y49E, R57E, G111D, M134E and G158E) by using the QuikChange lightning mutagenesis kit. The correct insertion of all mutations was verified by sequencing.

Upon cloning into pET25b, the recombinant vector was transformed into *E. coli* BL21(DE3), which was grown in LB media at 37 °C and 160 rpm with 100 mg/L ampicillin. Expression of 8M was induced via the addition of 0.5 mM isopropyl β-D-1-thiogalactopyranoside (IPTG) at 30 °C. After 10 hours, cells were harvested by centrifugation (12,000 rpm, 15 min at 4 ºC) and washed once with 20 mM sodium phosphate buffer, pH 7.8. The resulting cell pellet was resuspended in 10 ml/g cells of 20 mM sodium phosphate buffer, pH 7.8, and was disrupted by

sonication. The cell debris was removed by centrifugation at 12,000 rpm to obtain crude lysate. which were then subjected to 20-50% ammonium sulfate saturation. The precipitated proteins were collected by centrifugation (10,000 rpm, 15 min at 4 °C) and dissolved in 50 mM Tris-HCl buffer, pH 8.0, followed by dialysis against the same buffer. The dialyzed protein solution was filtered and loaded onto anion Q Sepharose column in 50 mM Tris-HCl buffer pH 8.0. The target protein was then eluted on a linear gradient of 0–1 M NaCl in the corresponding binding buffer. The collected active fractions were pooled and dialyzed extensively against 10 mM sodium phosphate buffer, pH 7.0 at 4 °C. The resulting samples were lyophilized extensively for long-term storage and synthesis of biofluid enzymes. Purity was examined by running 12% SDS-PAGE. Protein quantitation was performed by the BCA method[4].

Preparation of protein-polymer conjugates

Solvent-free liquid [c8M][S] and [cMb][S] was prepared as described previously[5,6], including cationization of the enzyme and subsequent electrostatic conjugation with the anionic polymer surfactants[7,8]. N,N'-dimethyl-1,3-propanediamine (DMPA) was coupled to the solvent-accessible carboxyl groups (C-terminal α-carboxylate, and Asp/Glu) of 8M or Mb via carbodiimide activation. 2 mL of a DMPA solution (2 M) was adjusted to pH 6 using 6 M HCl, and added dropwise with stirring to 10 mL of pure proteins (~1 mg/mL; 20 mM sodium phosphate buffer, pH 6.5). The coupling reaction was initiated by adding 200 mg of 1-ethyl-3-(3-(dimethylamino)propyl)-carbodiimide (EDC) immediately and again after 4 h. The pH value was maintained at 6.0 using 0.2 M HCl, and solutions were stirred for another 6 h. The solutions were centrifuged, filtered (VWR PES membrane, 0.2 μm), and dialyzed (MWCO = 12-14000 Da) against Milli-Q quality water for 48 h to produce stable solutions of the DMPA-modified proteins (called c8M or cMb); the resultant cationized protein solutions were added dropwise with stirring to anionic surfactant solutions (pH 6.8, 10 mg/mL)[2,7] at a protein charge:surfactant molar ratio of 1:3, and stirred overnight to produce aqueous solutions of nanoconjugates. The unbound surfactant molecules were removed by dialysis against Milli-Q quality water for 48 h, and the resulting aqueous solutions were then lyophilized for 48 h to produce white low-density powders, which yield white transparent solvent-free melts upon thermal annealing at 60 °C.

For the synthesis of solvent-free liquid [a8M][cS] and [aMb][cS], 8M and Mb were both succinylated first and then electrostatically conjugated with the cationic polymer surfactants.

Briefly, the lyophilized powder of 8M (or Mb) was resolved in 0.2 M sodium borate, pH 8.5 to prepare 10 mL of protein solutions (~2 mg/mL). Then 50 mg of succinic anhydride was dissolved in 1 mL of dry dioxane, followed by addition dropwise with stirring into the protein solution. The pH of the reaction mixture was maintained around 8.0 by periodic addition of 0.2 M NaOH. The reaction was continued overnight to ensure complete blocking of all surface amine groups. Subsequently, the solutions were centrifuged, filtered (VWR PES membrane, 0.2 μm), and dialyzed (MWCO = 12-14000 Da) against Milli-Q quality water for 48 h to produce stable solutions of the anionized enzymes (called a8M or aMb); the resultant anionized protein solutions were added dropwise with stirring to cationic surfactant solutions (pH 7.2, 10 mg/mL) but at a protein charge : surfactant molar ratio of 1:6 instead, and stirred overnight to produce aqueous solutions of nanoconjugates. The unbound surfactant molecules were removed by dialysis against Milli-Q quality water for 48 h, and the resulting aqueous solutions were then lyophilized for 48 h to produce white low-density powders, which yield white transparent solvent-free melts upon thermal annealing at 60 °C.

The molar ratio of protein to polymer was estimated by resolving the lyophilized samples with known weight with Milli-Q water followed by protein concentration assay with BCA method.

MALDI-TOF MS analysis

Samples were analyzed by MALDI-TOF Mass spectroscopy following a similar procedure previously described in the literature [9]. An aliquot (1-5 μl) of the derivatized proteins was lyophilized, resuspended in 2 μl 1% trifuoroacetic acid and mixed with 2 μl 2,5-dihydroxyacetophenone (0.1 M in 20 mM ammonium dihydrogen citrate and 75% (v/v) EtOH). The material (1 μl) was spotted onto a stainless steel target and allowed to dry. The spectra were recorded in positive and linear mode using an AutoFlex Smartbeam III instrument (Bruker) calibrated by external calibration (Peptide calibration standard I; Bruker Daltronics). The centroid masses determined were evaluated using the GPMAW software (www.gpmaw.com).

TNBS assay for free amino groups

In the modification of 8M, the number of free amino groups was determined by a TNBS (2,4,6-trinitrobenzene sulfonic acid) assay. TNBS can react with primary amines and generate a highly chromogenic product with strong absorbance at 335 nm[10]. Bovine serum albumin (BSA)

was used as the standard agent. Briefly, 100 μl protein solutions of a series of concentrations (in 0.1 M sodium bicarbonate, pH 8.5) was mixed well with 50 μL of 0.01% (w/v) TNBS solution in each well of a 96-well plate, and then incubated at 37 °C for 2 hours in a constant-temperature incubator, followed by the addition of 50 μl of 10% SDS and 25 μl of 1 N HCl to each sample. The absorbance values at 335 nm were then determined on a spectrophotometer. Simultaneously, protein concentration assays were performed with BCA method.

The absorbance values of the TNBS-treated BSA, native 8M and anionized 8M as a function of protein concentration were plotted and fitted as linear curves. Inspection of BSA crystal structure indicated that there are 60 free amine groups on BSA surface[11,12], hence the absorbance values of the TNBS-treated BSA could be plotted as a function of the concentration of free amines on BSA. Accordingly, the molar ratio of amines group of 8M or a8M to 8M or a8M could be calculated based on the plot.

Zeta potential measurement

Zeta potential measurements were performed on aqueous solutions (1 $mgmL^{-1}$, 20 mM sodium phosphate buffer, pH 6.8) using a Malvern Instruments Zetasizer Nano ZS (Malvern Instruments, Worcestershire, UK) at 25 °C. The mean values of three measurements were calculated.

UV-vis spectroscopy

UV/vis spectroscopy was performed on a Shimadzu UV2700 fitted with a water bath temperature control device. Aqueous solutions of Mb, cMb, aMb, [cMb][S] and [aMb][cS] were measured in 10 mm quartz cuvettes at 25 °C. Solvent-free liquid [cMb][S] and [aMb][cS] were measured in a 0.01 mm two-part quartz cell at 45 °C. All spectra were recorded between 700 and 300 nm.

Small-Angle X-ray Scattering

75 μl sample of either unbuffered aqueous protein or protein-surfactant conjugates of known protein concentration was loaded on the flux- and background optimized SAXS instrument at Aarhus University[13]. It is a NanoSTAR SAXS camera from Bruker AXS with a Cu rotating anode x-ray source with a wavelength of λ = 1.54 Å. The instrument uses a set of homebuilt scatterless slits in front of the sample[14]. Measurements were done for one hour at 20 °C and

water was used for background subtraction and calibration standard for absolute scale conversion. All SAXS data are plotted as a function of the scattering vector q given by $q = 4\pi \sin(\theta)/\lambda$. The Indirect Fourier Transformation (IFT) procedure[15] was used to gain model independent information on the protein-surfactant complexes. The home-written program WIFT[16,17] was used for obtaining the pair-distance distribution function ($p(r)$) which is a histogram of distances between pair of points weighted by the excess scattering length density at the points. This function thus gives information on real-space distances in the complexes. The $p(r)$ functions were normalized so the maximum value was 1.

Modelling of the data was done also using home-written programs. The native and modified 8M were fitted using the pdb structure of BsLA (pdb:1ISP[18]) while Mb was fitted using the crystal structure of Mb (pdb: 1MBN[19]). Fitting was done on absolute scale with the scattering calculated using the Debye equation[20] including hydration layer and with a background as fitting parameter. In some cases surface modifications caused unfolding of the protein and this was modelled as a random coil protein obeying Gaussian statics of Debye[21].

S and cS polymers have similar structures and only differ in their headgroups where the electron density is very similar. For this reason the same parameters were used for S and cS when describing the complexes or polymers. The micelles were modelled as that of a core-shell spherical structure with some polymer scattering arising from The PEG chains as described before for S polymer[22].

For the protein-surfactant conjugates, a core-shell model on absolute scale was used[22]. To decrease the amounts of fitting parameters, the core consisting of the protein, was keep constant with dimensions of that of the folded protein. To estimate the dimensions of folded 8M or Mb a oblate ellipsoid of revolution was used with radius r = 17.4 Å or r = 24.8 Å and eccentricity ε = 1.64 or ε = 0.56, respectively. In the model, the core is surrounded by a layer from the alkyl chain of S followed by a PEG layer described by a graded outer interface. Free micelles was used to describe polymer surfactant not bound to the protein as also done in a previous study[22] but further analysis showed that free micelles were not present in any of the samples. For cS polymer alone and for some complexes, repulsive interactions were observed in the scattering data as a decrease in intensity for low q, and a structure factor was included to address this as described before with a hard sphere radius (R_{hs}) and a volume fraction (η_{hs}) describing the interference effect[23].

Differential scanning calorimetry analysis

Differential scanning calorimetry (DSC) of the solvent-free liquid lipases was run between -70 °C and 70 °C at a scan rate of 10 °C/min on a Pyris 6 DSC system (Perkin-Elmer Cetus, Norwalk, USA). Approximately 5 mg of each lyophilized sample was put into an aluminum pan and placed in the equipment under a purging atmosphere of nitrogen. The DSC scans were evaluated using MicroCal Origin 8.6 software.

Attenuated total reflection Fourier transform infrared (ATR-FTIR)

ATR-FTIR was used for the characterization of lipase secondary structures. Samples were pressed onto a ZnSe ATR crystal mounted in a trough plate and placed in a horizontal ATR-FTIR spectrometer (Bruker Vertex 80V FTIR). The spectra were collected with an unpolarized beam at a resolution of 4 cm^{-1} with at least 64 scans, and a spectral window of 400–4000 cm^{-1}. Background spectra of the clean ZnSe crystal disc were collected at initial temperatures and subtracted from the sample spectra. The FTIR spectra were analyzed using MicroCal Origin 8.6 software.

Supplementary references

[1] J. Araki, C. Zhao, K. Ito, *Notes* **2005**, *3*, 7524.

[2] F. X. Gallat, A. P. S. Brogan, Y. Fichou, N. McGrath, M. Moulin, M. Härtlein, J. Combet, J. Wuttke, S. Mann, G. Zaccai, C. J. Jackson, A. W. Perriman, M. Weik, *Journal of the American Chemical Society* **2012**, *134*, 13168.

[3] Y. Zhou, B. Perez, W. Hao, J. Lv, R. Gao, Z. Guo, *Biochemical Engineering Journal* **2019**, *148*, 195.

[4] P. K. Smith, R. I. Krohn, G. T. Hermanson, A. K. Mallia, F. H. Gartner, M. D. Frovenzano, E. K. Fujimoto, N. M. Goeke, B. J. Olson, D. C. Klenk, *ANALYTICAL BIOCHEMISTRY* **1985**, *19*, 76.

[5] A. P. S. Brogan, G. Siligardi, R. Hussain, A. W. Perriman, S. Mann, *Chemical Science* **2012**, *3*, 1839.

[6] Y. Zhou, N. C. Jones, J. N. Pedersen, B. Pérez, S. V. Hoffmann, Søren Vrønning Petersen, J. S. Pedersen, A. Perriman, P. Kristensen, R. Gao, Z. Guo, *ChemBioChem* **2019**, *20*, 1266.

[7] A. P. S. Brogan, K. P. Sharma, A. W. Perriman, S. Mann, *Nature Communications* **2014**, *5*, 5058.

[8] A. W. Perriman, H. Cölfen, R. W. Hughes, C. L. Barrie, S. Mann, *Angewandte Chemie - International Edition* **2009**, *48*, 6242.

[9] T. Wenzel, K. Sparbier, T. Mieruch, M. Kostrzewa, *Rapid Communications in Mass Spectrometry* **2006**, *20*, 785.

[10] A. F. S. A. Habeeb, *Analytical Biochemistry* **1966**, *14*, 328.

[11] A. Bujacz, *Acta Crystallographica Section D: Biological Crystallography* **2012**, *68*, 1278.

[12] K. A. Majorek, P. J. Porebski, A. Dayal, M. D. Zimmerman, K. Jablonska, A. J. Stewart, M. Chruszcz, W. Minor, *Molecular Immunology* **2012**, *52*, 174.

[13] J. S. Pedersen, *Journal of Applied Crystallography* **2004**, *37*, 369.

[14] Y. Li, R. Beck, T. Huang, M. C. Choi, M. Divinagracia, M. C. C. & M. D. Y. Li, R. Beck, T. Huang, *Journal of Applied Crystallography* **2008**, *41*, 1134.

[15] O. Glatter, *Journal of Applied Crystallography* **1977**, *10*, 415.

[16] J. S. Pedersen, S. Hansen, R. Bauer, *European Biophysics Journal* **1994**, *22*, 379.

[17] C. L. P. Oliveira, M. A. Behrens, J. S. Pedersen, K. Erlacher, D. Otzen, J. S. Pedersen, *Journal of Molecular Biology* **2009**, *387*, 147.

[18] K. Kawasaki, H. Kondo, M. Suzuki, S. Ohgiya, S. Tsuda, *Acta Crystallographica Section D: Biological Crystallography* **2002**, *58*, 1168.

[19] Ha. C. Watson, *Prog Stereochem* **1969**, *4*, 229.

[20] P. Debye, *Ann Phys-Berlin* **1915**, *45*, 809.

[21] P. Debye, *J. Phys. Colloid Chem.* **1947**, *51*, 18.

[22] B. Pérez, A. Coletta, J. N. Pedersen, S. V. Petersen, X. Periole, J. S. Pedersen, R. B. Sessions, Z. Guo, A. Perriman, B. Schiøtt, *Scientific Reports* **2018**, *8*, 12293.

[23] J. S. Pedersen, *Physical Review B* **1993**, *47*, 657.
